# Are we working (too) comfortably?: the systematic development of an intervention to support workers to move more while working at home

**DOI:** 10.1186/s12966-025-01762-3

**Published:** 2025-06-23

**Authors:** Divya Sivaramakrishnan, Claire Fitzsimons, Sarah Morton, Jillian Manner, Ruth Jepson, Ailsa Niven

**Affiliations:** 1 Scottish Collaboration for Public Health Research and Policy, University of Edinburgh, Edinburgh, UK; 2 Physical Activity for Health Research Centre, University of Edinburgh, Edinburgh, UK

**Keywords:** Sedentary behaviour, Work from home, Occupational health, Desk-based employees, Intervention development

## Abstract

**Background:**

Covid-19 accelerated a transformational change in working practices, with a considerable proportion of desk-based workers now engaged in home or hybrid working. Working at home appears to exacerbate the already elevated levels of unhealthy occupational sedentary behaviour, and there is a need to support employees to reduce sedentary behaviour while working at home. The aim of this study was to develop an intervention to support employees to reduce sedentary behaviour when working at home utilising the novel integration of the Six Steps in Quality Intervention Development (6SQuID) and Behaviour Change Wheel (BCW) intervention development frameworks.

**Methods:**

We report on 6SQuID steps 1–5, with the Capability, Opportunity and Motivation influence on Behaviour (COM-B) model integrated into steps 1–4, providing a theoretical organising framework. In step 4, the intervention functions and behaviour change technique elements of the BCW were used to accurately and consistently specify the active ingredients of the intervention. The test and refine phase of 6SQuID (step 5) evaluated the acceptability of elements of the intervention with a sample of Scottish Government employees.

**Results:**

The causal factors for employee sedentary behaviour while working at home were delineated, and theory of change/action models were constructed. Intervention components were developed to address causal factors and presented as a toolkit on an online platform. These comprised: 1) education on sedentary behaviour, 2) resources to aid intention formation and action planning, 3) strategies to increase movement, and 4) suggestions for support from colleagues/friends/family. Strategies aimed at line managers and organisations to support employees and create an organisational culture that enables employees to move more were also developed. The acceptability testing demonstrated the value of the toolkit format incorporating a suite of strategies, and feedback informed refinement of the toolkit.

**Conclusions:**

This study addresses the urgent need to support employees to reduce sedentary behaviour while working at home. Using the novel application of integrated intervention development frameworks, a comprehensive intervention toolkit has been formulated and preliminarily tested. The toolkit comprises strategies and resources for employees, line managers and organisations. Further feasibility and effectiveness testing with a larger sample is recommended prior to large-scale implementation.

**Supplementary Information:**

The online version contains supplementary material available at 10.1186/s12966-025-01762-3.

## Introduction

Sedentary behaviour (SB) is defined as any waking behaviour characterised by an energy expenditure ≤ 1.5 metabolic equivalents (METs), while in a sitting, reclining or lying position [1]. High levels of SB are associated with increased risk of cardiovascular and all-cause mortality [2], Type 2 Diabetes, cardiovascular disease [3], musculoskeletal health issues [4], and poor mental health [5]. Physical activity can modify the associations between health risks and SB [6], with the most recent evidence indicating that physical activity of any intensity (albeit of different duration) can attenuate the association with all-cause mortality [2]. Consequently, it is recommended that a dual approach to reducing SB is adopted by adults to both limit the amount of sedentary time and to replace it with physical activity of any intensity for better health outcomes [3, 7].

The office environment is a high risk setting for elevated SB [8, 9], with sedentary time among desk-based employees reported to be up to 82% of their working day [10–12]; equivalent of up to 428 min/day [12]. Consequently, there has been a growth in intervention research to support employees to reduce SB when at work (e.g., [13]). Whilst this body of research provides valuable insight into how to support employees to reduce SB in the office setting, the Covid-19 pandemic has led to a transformational change in working practices that requires consideration. Covid-19 lockdown restrictions necessitated a shift from the office to homeworking for many employees, and this shift has initiated an enduring change in work patterns with an increase in home and hybrid (i.e., spending a proportion of the working week at home) working. For example, UK data indicate that pre-Covid just 12% of the working population had worked at home on at least one day in the previous 7-days [14], and post-Covid this is now around 40% (November/December 2024; [15]), with 13% of respondents working only from home. Unfortunately, this shift to home working appears to have exacerbated already high levels of occupational SB evident in office workers, both during [16, 17] and post-Covid-19 [18, 19]. To date, there has been limited intervention research on supporting workers to reduce SB when working at home, with one study demonstrating the benefits of height-adjustable desks and an online programme both individually and in combination to reduce sitting time [20]. Nevertheless there is growing interest in this issue, with a recent programme of research reporting on preliminary development work [21, 22] and the protocol for a RCT evaluation [23] of a new digital intervention called Click2Move to reduce home workers sedentary behaviour in several European countries.

Supporting employees to reduce SB while working at home requires a complex intervention that considers the nature of the behaviour, population, environment, and culture [24]. The Medical Research Council (MRC) Framework for Developing and Evaluating Complex Interventions [25] highlights intervention development as a distinct step. There is a plethora of health intervention development approaches with O’Cathain et al. [26] identifying six different categories of approaches. Within occupational SB intervention development, researchers have utilised a range of approaches to design interventions (e.g., [27]), with three recent studies drawing on the Behaviour Change Wheel (BCW; [28]) [23, 29, 30]. In our initial intervention development work focusing on reducing SB when working from home [16, 24, 31], we also used the BCW. In short, the BCW outlines a series of stages to intervention development including understanding the target behaviour and what needs to change using the theoretical Capability Opportunity Motivation-Behaviour (COM-B) framework, identifying intervention options, and establishing the intervention content in terms of behaviour change techniques (BCTs), and how these should be delivered [28]. A strength of the BCW approach is the strong theoretical underpinnings of behaviour, and the focus on enhancing the accuracy and quality of intervention descriptions through the use of common language for intervention functions and their active components of interventions (i.e., BCTs). However, there are limitations to the BCW. Firstly, the COM-B framework does not fully address the broader systems-level influences on behaviour [32], such as organisational culture in the workplace setting [33]. Secondly, the BCW approach does not specify the value of articulating a detailed programme theory outlining how the intervention is expected to achieve the desired outcomes through specified activities [34]. Programme theories are a critical part of intervention development to help communicate how the intervention is expected to work and inform future outcome and process evaluation [35]. Consistent with the BCW approach, none of the previously developed interventions [23, 29, 30] outlined a detailed programme theory in their development of interventions targeting occupational sedentary behaviour, although Bort-Roig et al. [23] detailed a logic model, and a logic model from *Stand More At Work* [29] was detailed in a later outcome evaluation paper [36].

An intervention development framework that addresses both the wider context and the development of programme theories is the Six Steps in Quality Intervention Development framework (6SQuID; [34, 37]. Tirman et al. [38] used 6SQuID in developing the ‘*Stand Up for Health*’ intervention to reduce SB in contact centres (i.e., organisations that manage customer interactions employing multiple communication channels including phone, online chat and email). They outlined a comprehensive programme theory detailing a theory of change (mechanism by which the change is expected to occur) and theory of action (how the intervention is constructed or the activities that are designed to trigger or activate the change mechanism) [38]. However, a limitation of this approach was restricted integration of detailed behaviour change theory evident in other interventions [29, 30]. Further, whilst Tirman et al. [38] described in detail the proposed intervention, integration of common intervention language regarding the intervention type and specific behaviour change techniques was not included, and would be beneficial in future work to facilitate consistency in reporting and fidelity of implementation.

The aim of this study was to adopt an original approach to intervention development by integrating the BCW and 6SQuID approaches to build on the strengths and address the limitations of each. Through this rigorous process, a novel evidence-informed intervention was systematically developed to support the substantial number of individuals who are working at home to reduce their SB.

## Method

The development of the intervention was structured around the six steps of the 6SQuID framework [34, 37]: Step 1. Understand the problem and its causes; Step 2. Identify modifiable causal factors; Step 3. Identify how to bring about change: theory of change; Step 4. Identify how to deliver change mechanisms: theory of action; Step 5. Test and refine the intervention; and Step 6. Collect sufficient evidence of effectiveness to proceed to a rigorous evaluation. We report on the first five steps. At this stage of reporting, Step 6 has not yet been undertaken.

In steps 1–4, the COM-B model from the BCW [28] was integrated to provide a theoretical framework for organising the factors that influence SB. Additionally, in step 4, we used the intervention functions and BCT elements of the BCW to accurately and consistently specify the purpose and active ingredients of the intervention. Coproduction with stakeholders and partners during intervention development is likely to enhance acceptability and feasibility of the intervention [34, 37]. The current study was informed by the principles of coproduction at every stage, through working with stakeholders who have a remit for workplace health in Scotland. Partners were consulted on methodological aspects and findings, and joined regular workshops where the project team shared progress and procured feedback.

**Fig. 1:**
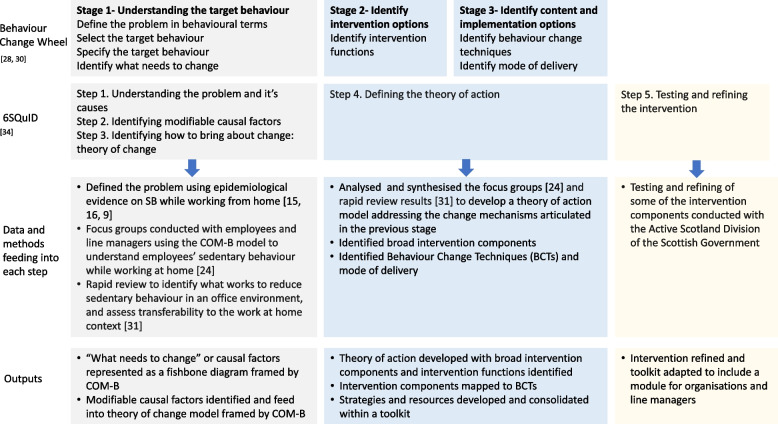
Integration of Behaviour Change Wheel and 6SQuID and methods and output for each stage

This intervention development process included activities that have previously been reported in detail elsewhere, including a descriptive cross-sectional study [16], rapid review [31], focus group study [24], and small-scale evaluation [39]. In this paper, these studies are referred to within the appropriate steps of the 6SQuID to comprehensively detail the intervention development. The integration of the BCW model and the 6SQuID framework and the methods informing each step are presented in Fig. 1.

### Step 1. understand the problem and its causes

This step identified (i) what is the problem and why we need to develop an intervention to address it and (ii) the causes or factors influencing the problem. The epidemiological evidence relating to health risks of SB are well-established [3]. This step was informed by findings from the descriptive cross-sectional study [16] and a focus group study with employees and line managers exploring the factors influencing SB while working at home [24]. From these studies, the hypothesised causal factors of employee SB while working at home were consolidated for each dimension of the COM-B model. The causal factors relating to employee and line mangers’ behaviour in supporting employees to reduce SB while working from home were then depicted using a fishbone diagram (a cause-and-effect diagram used to identify possible causes of a complex problem) [34].

### Step 2. identify modifiable causal factors

Step 2 involved identifying which of the causal factors relating to capability, opportunity and motivation are potentially modifiable (i.e., factors that can be changed or controlled) or non-modifiable/hard to modify [34]. This informed identification of causal factors that the intervention should target to influence the behaviour [34]. Those factors described in the fishbone diagram (step 1) were categorised as modifiable or non-modifiable/hard to modify.

### Step 3. identify how to bring about change: theory of change

In step 3, a theory of change was outlined, which aimed to describe the hypothesised mechanisms that will bring about change, and visually represented as a logic model. The COM-B model [28] was considered central to the theory of change for this study and underpinned the studies contributing to its development [16, 24]. The theory of change logic model was constructed linking the factors identified using the COM-B model (depicted in the fishbone diagram) to a chain of outcomes (short, medium and long-term). In an occupational setting, the target of reducing SB can have several long-term benefits for the employee as well as for the organisation including physical and mental health outcomes and work performance [4, 38, 40]. The logic model was constructed to include hypothesised long-term outcomes. The theory of change model developed in this step demonstrated how the identified causal factors rooted in the COM-B model lead to short-, medium- and long-term outcomes.

### Step 4. identifying how to deliver change mechanisms: theory of action

The theory of action refers to the specific activities or interventions components that would trigger the change mechanisms specified in the theory of change [34]. In this step, the change mechanisms identified in the previous step were considered and intervention activities to address them were developed. While developing an intervention, the MRC framework recommends considering if an existing intervention could be adapted to a new context [25]. The shift to home working was accelerated by the Covid-19 pandemic [15], and SB research in the home-environment is still nascent. However, high quality research on office-based SB interventions is available, and in order to draw from this well-developed evidence base, a rapid review was conducted to identify interventions with a beneficial effect in reducing SB in the office environment, followed by an assessment of transferability to the home-working environment [31]. Through the rapid review and transferability assessment, potential intervention strategies to reduce SB in the home environment were identified and fed into the step 4 of intervention development. The theory of action mapped during this step shows the intervention components developed to address the identified change mechanisms, with each component comprising of a range of strategies and resources.

To further define the intervention content, we used the BCW framework to specify the intervention functions and Behaviour Change Techniques (BCTs) [28]. This approach allows precise details of the content of an intervention to be reported using a common language. The intervention functions are the broad categories by which an intervention can change behaviour and were selected from the nine possible intervention functions in the BCW (i.e., education, persuasion, incentivisation, coercion, training, restriction, environmental restructuring, modelling and enablement). A BCT is defined as “a replicable component of an intervention designed to alter or redirect causal processes that regulate behaviour” ([41] p.694). It should be the smallest component that can be regarded as an ‘active ingredient’ within the intervention and can be used alone or with other BCTs. For this work we aligned with the Behaviour Change Technique Taxonomy v1 (BCTTv1) with 93 BCTs clustered into 16 groupings [42]. The mapping process was carried out by members of the research team (AN, CF, SM and DS).

### Step 5. testing and refining the intervention

The 6SQuID model frames testing of the intervention as an iterative process, involving pilot and feasibility studies to understand if the activities identified (step 4) are feasible and work as intended [34]. Testing can be carried out at any stage during the intervention development process and can take the form of testing distinct intervention components. In step 5, we undertook small-scale testing to consider feasibility of delivery, participant engagement and acceptability of some of the intervention components, in collaboration with the Active Scotland Division of The Scottish Government during August to December 2022 (testing Oct to Nov 2022) [39]. We received institutional ethical approval (SMOR03102022) to collect evaluative research data. Desk-based employees of The Scottish Government who spent some of their working week at home were recruited via an optional webinar for a specific department, and an in-house whole staff blog. Both the webinar and blog included educational information (e.g., information in relation to SB and health; elevated SB in the home environment), details of the research, and an invitation to participate by contacting one of the research team (SM). The intervention was delivered through email newsletters (using DotDigital) delivered weekly for four weeks. Each newsletter had a theme (e.g., Active Meetings weeks) and was received on a Monday morning, allowing participants to plan and integrate the strategies across the course of their working week. Participant engagement was evaluated by examining metrics relating to attendance at the webinar, views of the blog, sign-ups to the intervention, and the percentage of the newsletter recipients who opened each weekly email. Additionally, participants’ feedback on the acceptability of the interventions, preferred weekly theme, and responses to individual statements relating to the perceived benefits of participation (e.g., more productive, mood is more positive, greater job satisfaction) were collected via an online evaluation questionnaire at the end of the intervention. These findings ‘looped back’ to inform further refinement of the final intervention.

## Results

The findings and outputs from the analysis for each step of the 6SQuID framework are presented below.

### Step 1. understanding the problem and it’s causes

#### What is the problem and why we need to develop an intervention to address it

As articulated in the introduction section, there is clear evidence of an increase in home working since Covid-19 [15], and indication that home working may exacerbate already elevated levels of SB. Daily levels of SB while working at home was reported to be as high as 89% in 2021 [16], compared to 60%−79% reported in office-based studies pre Covid [9]. SB is associated with several physiological and psychological health risks [3], and this study aims to address the significant public health problem of SB while working at home.

#### The causes or factors influencing the problem depicted using a fishbone diagram

The target behaviour was SB while working at home, and the definition is consistent across all the studies that have informed the intervention development process [16, 24, 31].

**Fig. 2:**
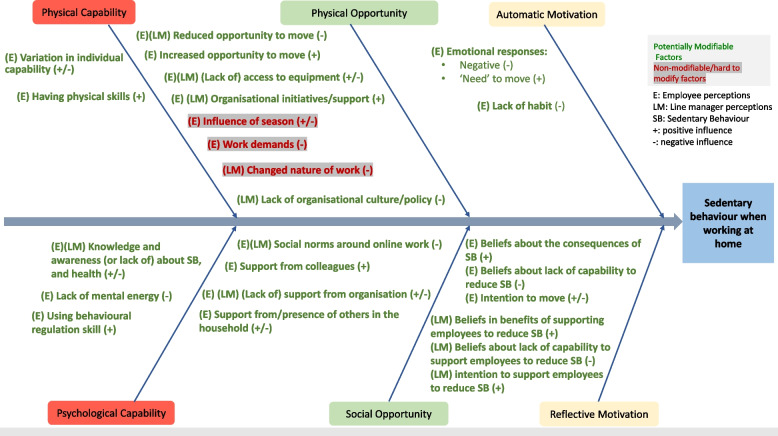
Fishbone diagram of the causal factors influencing employee SB while working at home

Niven et al (2023) demonstrated that there were significant differences in how capability, opportunity and motivation factors influenced sitting behaviour and provided initial evidence to explore factors influencing SB using a COM-B lens. Findings from the focus group study [24], conducted with employees and line managers, were used to identify hypothesised causal factors for employee SB while working at home. The study captured employees’ perceptions on SB and line managers’ perceptions on supporting employees to reduce SB [24]. The results informed the development of a fishbone diagram, depicting the causal factors organised by the COM-B dimensions (Fig. 2).

### Step 2. identifying modifiable causal factors

The causal factors influencing SB while working at home outlined in the fishbone diagram were considered by the research team and categorised as potentially modifiable (coded as green in Fig. 2), and non-modifiable/hard to modify (coded as red in Fig. 2). Most factors were categorised as modifiable. Three factors under physical opportunity were deemed to be non-modifiable or hard to modify. These included i) seasonal and weather-related influences ii) work demands and iii) changed nature of work (e.g., work not being suitable for moving meetings due to the requirement for screens to share information). The factors identified as modifiable informed step 3 of the intervention development process.

### Step 3. identifying how to bring about change: theory of change

**Fig. 3:**
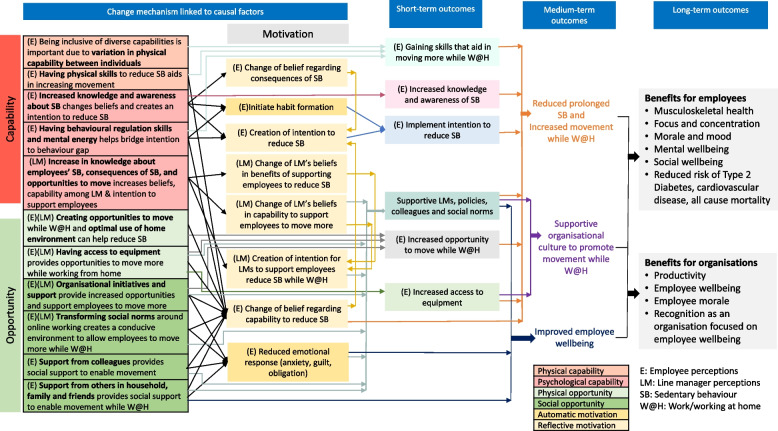
Theory of change logic model

The theory of change logic model (Fig. 3) that was developed shows potential, hypothesised mechanisms of change linked to short, medium and long-term outcomes. The mechanisms of change are grounded in the causal factors identified in steps 1 and 2, framed by the COM-B model [28]. The COM-B model conceptualises opportunity and capability as influencing behaviour both directly and by influencing motivation factors [28], and this is reflected in the theory of change logic model (Fig. 3). The capability (physical and psychological) and opportunity (physical and social) factors influence motivation (reflective and automatic), and all the causal factors are linked to the chain of outcomes. Some capability and opportunity factors directly lead to short- and medium-term outcomes (e.g., being inclusive of diverse capabilities leads to the short-term outcome of employees gaining skills that aid in moving more while working at home). Other factors may influence motivation that then influences outcomes, and may also be directly linked to outcomes. For example, having physical skills to reduce sedentary behaviour (physical capability) may change employee beliefs around capability to reduce sedentary behaviour (reflective motivation), and also leads to the short-term outcome of gaining skills that aid in moving more while working from home. Similarly, aspects of motivation may have both direct effects on outcomes, and indirectly through reinforcing other aspects of motivation (e.g., change in beliefs leads to creation of intention).

The employee capability factors (i.e., being inclusive of diverse capabilities; having physical skills to reduce SB; increased knowledge of SB and its consequences as well as awareness about their own levels of SB; having behavioural regulation skills and mental energy) influence motivation factors such as change of belief regarding consequences of sedentary, change of beliefs around capability to reduce sedentary behaviour, creation of an intention to reduce sedentary behaviour and initiating habit formation (Fig. 3). These lead to the short-term outcomes of gaining skills that aid in moving more while working from home, increased knowledge and awareness of sedentary behaviour, and implementing the intention to reduce sedentary behaviour. Among line managers, knowledge about employees’ sedentary behaviour, consequences of sedentary behaviour, and opportunities to move leads to changes in motivational factors such as line managers’ beliefs regarding benefits of supporting employees to reduce sedentary behaviour, beliefs around capability to support their employees, and creates an intention for line managers to support employees to move more while working from home.

Creating opportunities to move more while working from home and having access to equipment (physical opportunity) change employee beliefs around capability to reduce sedentary behaviour (reflective motivation), and lead to the short-term outcome of increased opportunities to move while working at home (Fig. 3). Organisational initiatives and support, transforming social norms around online working, support from employees and support from others in the household/friends/family are social opportunity factors that are hypothesised to change employee beliefs regarding capability to reduce sedentary behaviour (reflective motivation) and target feelings of anxiety, guilt and obligation (automatic motivation). All of these factors, as well as factors influencing line manager capability, lead to the short-term outcome of having supportive line managers, policies, colleagues and social norms.

Three medium-term outcomes were identified including (i) reduced prolonged sedentary behaviour and increased movement among employees while working at home (ii) supportive organisational culture to promote movement while working at home and (iii) improved employee wellbeing. Over time, these medium-term outcomes would lead to a host of longer-term benefits for both the organisation and employees (Fig. 3).

### Step 4. defining the theory of action and mapping behaviour change techniques

Intervention components were developed to address and activate the change mechanisms identified in step 3 (Fig. 3). Twelve broad intervention components were developed addressing capability and opportunity factors (and linked motivation factors). Each broad intervention component comprises a range of strategies, resources and suggestions (see Supplementary file 1 for detailed intervention components and Supplementary file 2 for Template for Intervention Description and Replication- TIDieR checklist). While the target behaviour is sedentary behaviour, the strategies and resources deliberately use inclusive language to avoid ableist framing [43] and encourage employees with a range of physical capabilities to “move more”. This also reflects the dual approach to reducing SB, aiming to limit the amount of sedentary time and replace it with increased levels of movement.

**Fig. 4:**
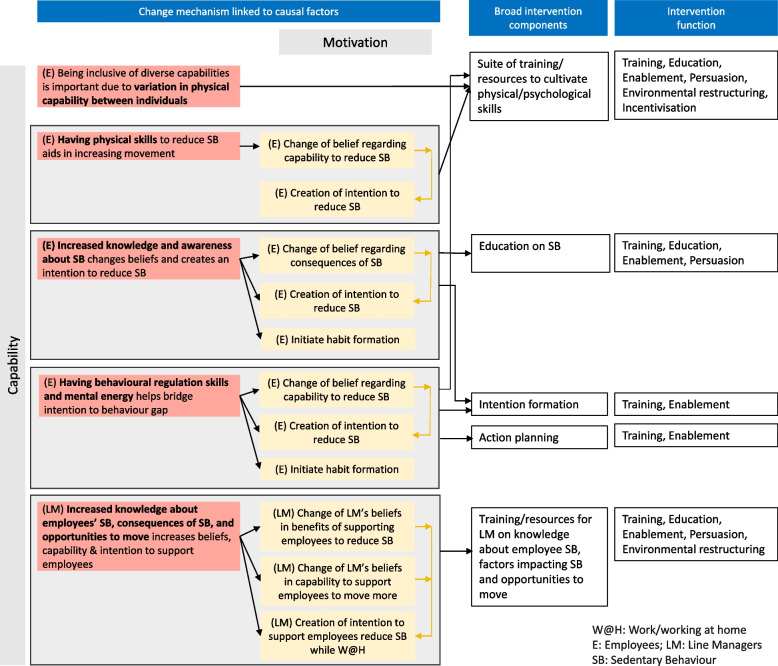
Theory of action- capability factors

Five broad intervention components were developed to address the causal mechanisms linked to capability (and in turn to motivation) (Fig. 4). These include developing a suite of training materials, and resources to cultivate physical and psychological skills, education on sedentary behaviour to increase employees’ knowledge and awareness, and resources to aid action planning and intention formation. Training and resources for line managers about employees’ SB, factors impacting SB and opportunities to move were also developed.

**Fig. 5:**
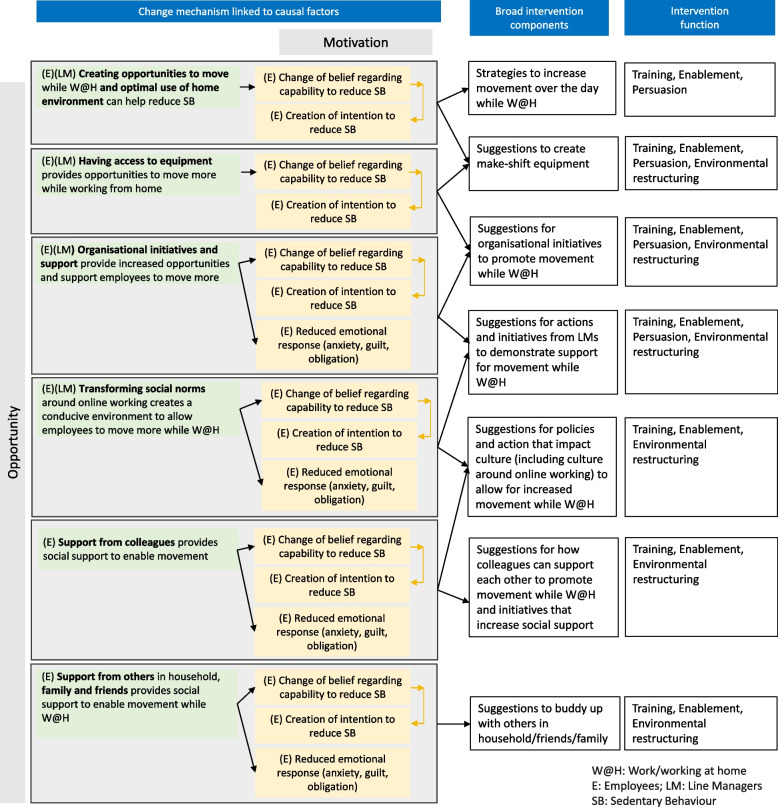
Theory of action- opportunity factors

Broad components to address opportunity (and linked motivation) factors (Fig. 5) include strategies for employees to increase movement over the day while working at home, suggestions to create make-shift equipment, suggestions for organisational initiatives to promote movement among employees while working at home, suggestions for actions and initiatives to enable line managers to demonstrate support for movement, suggestions for policies and action that impact culture to allow for increased movement while working at home, suggestions for how colleagues can support each other to promote movement and initiatives that increase social support, and suggestions to buddy up with others in household/friends/family.

In order to articulate the intervention comprehensively and enhance replicability, the detailed intervention components have been mapped to intervention functions and Behaviour Change Techniques (BCTs) (Supplementary file 1). Training, education, enablement, persuasion and environmental restructuring are the common intervention functions to address capability (and linked motivation factors) (Fig. 4). The common intervention functions to target opportunity (and linked motivation factors) are training, enablement, persuasion and environmental restructuring (Fig. 5). The BCTs identified, aggregated for the broad intervention components are presented in Table 1.

**Table 1:** Behaviour change techniques mapped to each broad intervention component

Broad intervention component		
Suite of training/resources to cultivate physical skills among employees	1.1 Goal setting behaviour 1.4 Action planning 2.2 Feedback on behaviour 4.1 Instruction on how to perform the behaviour 5.1 Information about health consequences 6.1 Demonstration of the behaviour 7.1 Prompts/cues 8.2 Behaviour substitution	8.3 Habit formation 8.4 Habit reversal 9.1 Credible source 10.7 Self incentive 12.1 Restructuring physical environment 12.5 Adding objects to the environment 13.1 Identification of self as role model
Suite of training/resources to cultivate psychological skills among employees	1.1 Goal setting behaviour 1.4 Action planning 2.2 Feedback on behaviour 4.1 Instruction on how to perform behaviour 6.1 Demonstration of behaviour 7.1 Prompts/cues	8.2 Behaviour substitution 8.3 Habit formation 8.4 Habit reversal 9.1 Credible source 12.5 Adding objects to the environment
Education on SB	2.2 Feedback on behaviour 4.1 Instruction on how to perform the behaviour 5.1 Information about health consequences	5.2 Salience of consequences 9.1 Credible source 8.7 Graded tasks
Intention formation	1.1 Goal setting behaviour 1.4 Action planning 4.1 Instruction on how to perform the behaviour	
Action planning	1.1 Goal setting behaviour 1.4 Action planning 4.1 Instruction on how to perform the behaviour	
Training/resources for LM on knowledge about employee SB, factors impacting SB and opportunities to move	1.1 Goal setting 1.2 Problem solving 1.4 Action planning 4.1 Instruction on how to perform the behaviour 5.1 Information about health consequences 5.2 Salience of consequences 5.3 Information about social and environmental consequences	9.1 Credible source 12.1 Restructuring physical environment 12.2 Restructuring social environment 12.3 Avoidance/reducing exposure to cues for the behaviour 13.1 Identification of self as role model
Strategies to increase movement over the day while W@H	4.1 Instruction on how to perform the behaviour 6.1 Demonstration of the behaviour 8.2 Behaviour substitution	9.1 Credible source 10.9 Self-reward
Suggestions to create make-shift equipment	4.1 Instruction on how to perform the behaviour 6.1 Demonstration of the behaviour 9.1 Credible source 8.2 Behaviour substitution	12.1 Restructuring the physical environment 12.5 Adding objects to the environment
Suggestions for organisational initiatives to promote movement while W@H	1.2 Problem solving 4.1 Instruction on how to perform the behaviour 12.2 Restructuring the social environment 13.1 Identification as self as role model	
Suggestions for actions and initiatives from LMs to demonstrate support for movement while W@H	1.2 Problem solving 4.1 Instruction on how to perform the behaviour 12.2 Restructuring the social environment 13.1 Identification as self as role model	
Suggestions for policies and action that impact culture (including culture around online working) to allow for increased movement while working from home	1.2 Problem solving 4.1 Instruction on how to perform the behaviour 12.2 Restructuring the social environment	
Suggestions for how colleagues can support each other to promote movement while W@H and initiatives that increase social support	1.2 Problem solving 3.1 Social support (unspecified) 4.1 Instruction on how to perform the behaviour 12.2 Restructuring the social environment	
Suggestions to buddy up with others in household/friends/family	3.1 Social support (unspecified) 4.1 Instruction on how to perform the behaviour 12.2 Restructuring the social environment	

*W@H* Work/working at home, *LM* Line Managers, *SB* Sedentary Behaviour

Most intervention components are now presented as a toolkit titled “*Move your way during the W@H day*” which is hosted on an online platform [44]. This mode of delivery was chosen to cater to the preference for “drip feeding” through a phased delivery of information, expressed by focus group participants [21]. The toolkit comprises five modules. Four of the modules have been developed for employees, and are organised into four weeks:

- Week 1: ‘Move More’ Meetings: includes animated videos and resources to encourage movement during meetings
- Week 2: Active Breaks Week: includes animated videos and resources to encourage active breaks
- Week 3: Active Commute Week: includes animated videos and resources with the suggestion to add a'home-to-home commute’ to the working day
- Week 4: DIY tech Week: DIY tech-based prompts suggestions using computer reminders, smartphone and smartwatch

Based on stakeholder feedback, a fifth module (titled ‘Guidance for managers and leaders to help workers move more during the work at home day’) was subsequently developed for managers and leaders, but has not yet been tested. One intervention component is currently delivered separately to the online toolkit (Reminders to move- Prompts delivered via MS Teams) and comprises a series of regular movement prompts delivered through MS Teams (development, testing and roll out will be reported elsewhere).

### Step 5. testing and refining the intervention

In step 5, components of the intervention were tested through delivery of four-weeks of email newsletters with Scottish Government employees (Active Scotland Division) [39]. Initial interest in the webinar and blog was very good, and elevated in comparison with other in-house activities (e.g., blog was accessed > 1000 times in 5 weeks). Initial sign-ups (*n* = 96) and engagement in the intervention was good, but reduced over time (e.g., 55% of sign-ups engaged in newsletter at week 1 and reduced to 22% by week 4). Feedback was generally positive, with participants reporting the email newsletters were a useful reminder to move more while working at home, and the toolkit strategies were practical without requiring additional costs or resource to implement. Active breaks was the most popular week. Notably 50% of final respondents (*n* = 18) reported improvements in mood, and more than a quarter of final respondents reported being more productive, and functioning better at work.

Suggestions for improvement of the intervention included providing detailed instructions on movement such as stretches, and subsequently short stretch videos were created and added to the final toolkit. Additionally, both participants’ feedback and the reduced engagement indicated the importance of enhancing organisational endorsement and ongoing leadership involvement to provide ‘permission’ and support for engagement with the intervention, and for senior staff to lead by example. Based on these observations and in line with the theory of change, a module to guide organisations and line managers was added to the final toolkit to address these points.

## Discussion

This study addresses an under-researched public health issue and describes the comprehensive development of an intervention to support the increasing number of workers in the UK and internationally who are working at home to reduce SB and move more. Although home or hybrid working is the new norm for many [15], research on supporting employees to reduce SB in the home environment is sparse. This programme of research makes an important contribution to the field in two ways. Firstly, the rigorous intervention development process adopted in this study was guided by the MRC Framework for Developing and Evaluating Complex Interventions [25] and takes a novel approach of integrating and leveraging the strengths of the Behaviour Change Wheel [28] and 6SQuID frameworks [34, 37] providing a unique methodological contribution to intervention development literature generally, and sedentary behaviour specifically. Secondly, this study consolidates findings from a robust body of published work undertaken by the project team [16, 24, 31, 39], and a complex intervention has been presented. The intervention comprises a suite of strategies to cater to different individual needs, preferences, capabilities, and environmental and social contexts. It allows for flexibility and can be implemented in organisations across varied sectors and sizes, and at a low cost. The intervention is curated on an online platform [44], with separate modules for employees and line managers. This intervention is an important contribution to the area, as only two existing interventions to support moving more in the home working environment have been identified [20, 23].

Through the rigorous intervention development process, several additional important outputs have been developed and specified that make a significant contribution to the field. These include a theory of change, theory of action, intervention functions, and BCTs. These outputs will be beneficial for both intervention implementation and outcome and process evaluation. In addition, specifying a theory of change and identifying the intervention functions allows for future adaptation of the intervention where the theory of action and mode of delivery can change in response to feedback, but stay true to the theory of change and address the causal factors [34]. This could also allow for the development of other interventions in different workplace contexts. For example, by using the same theory of change and developing a new theory of action.

Multi-level programmes have been found to be effective in reducing sedentary behaviour in office-based interventions [30, 36, 45, 46], and this premise also informed the newly developed Click2Move intervention [23]. In line with this evidence and informed by the developmental work, the current intervention encompasses the COM-B dimensions and has intervention components relating to capability, opportunity and motivation [28]. As noted previously, the COM-B model does not fully address systems-level influences on behaviour [32] and indeed the broader concept of organisational culture emerged as an important influence on behaviour from both the focus group study [24], and also noted by participants in the small scale study conducted with Scottish Government employees (see step 5) [39]. Norms around online working, flexible working arrangements, and support from the line manager were noted as factors that impacted movement while working at home. This is in agreement with recent studies emphasising the importance of considering organisational culture while designing workplace programmes [22, 33]. Recommendations to foster a positive workplace culture from Manner et al. [33] include role modelling by leaders, effective communication and taking into account the needs of the employees, and providing training and support to middle level managers, so that they can in turn support their staff. These recommendations were integrated into the toolkit, and especially Module 5.

### Strengths and limitations

Methodological strengths of this programme of research include the novel integration of the BCW and 6SQuID frameworks, transparency with respect to description of the intervention development process, and adoption of coproduction principles. As a result, the study has a strong theoretical foundation, considers individual and organisational contexts, and specifies a programme theory. The resultant toolkit offers a low cost, flexible resource for workplaces to adapt to both individual and workplace needs. A limitation is that several relationships articulated in the theory of change and theory of action are hypothetical. Although they are evidence informed, rigorous testing is required to confirm that the intervention works as intended, to further refine the intervention, and capture any unintended consequences.

### Future research

The next step recommended by the MRC [25] and 6SQuID [34] frameworks following the development of the intervention is feasibility testing. Whilst initial work on assessing the feasibility of some intervention components has been presented, testing the acceptability of the intervention and feasibility of implementation as well as evaluation design, using rigorous study designs, is required. It is recommended that an evaluability assessment is conducted before the feasibility trial commences, where the outcomes and study designs are discussed and agreed in collaboration with partners [25, 34]. The MRC Framework also suggests that economic modelling be undertaken at this stage to assess costs and benefits of the intervention including research costs [25]. While the mode of delivery adopted in the current study is a toolkit hosted on an online platform, it is important to consider and test other delivery modes such as a dynamic App, which could influence adoption and sustainability. Feasibility testing (continued step 5, 6SQuID) will inform refinement of the theory of change, identify unintended consequences, test acceptability of and recruitment to the intervention and mode of delivery, and justify a larger-scale effectiveness trial.

## Conclusions

This study makes an important contribution to the field of workplace health where home and hybrid working is the new norm. It also highlights the value of a rigorous intervention development process and advocates for the integration of systematic frameworks to ensure a strong theoretical foundation, comprehensive understanding of the behaviour and associated causes, and specification of a programme theory illustrating the intended intervention mechanisms. The resultant toolkit can be incorporated by organisations into wellbeing strategies to support employees who are working at home.

## Supplementary Information

Supplementary Material 1. Broad and detailed intervention components mapped to Behaviour Change Techniques (BCTs).

Supplementary Material 2. The TIDieR (Template for Intervention Description and Replication) Checklist.

## Data Availability

No datasets were generated or analysed during the current study.

## Abbreviations

SB: Sedentary Behaviour
MRC: Medical Research Council
BCW: Behaviour Change Wheel
COM-B: Capability Opportunity Motivation-Behaviour
BCT: Behaviour Change Technique
6SQuID: Six Steps in Quality Intervention Development
W@H: Work/Working at Home
LM: Line manager
