# Supplementary material for: Are we working (too) comfortably?: the systematic development of an intervention to support workers to move more while working at home
Source: Int J Behav Nutr Phys Act. 2025 Jun 23;22:84. doi: 10.1186/s12966-025-01762-3 (PMC12183816; doi:10.1186/s12966-025-01762-3)
Supplement: Supplementary file 2 — Supplementary Material 2. The TIDieR (Template for Intervention Description and Replication) Checklist. [file 12966_2025_1762_MOESM2_ESM.docx]

**
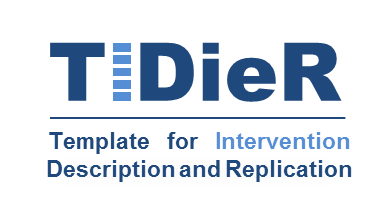
The TIDieR (Template for Intervention Description and Replication) Checklist*:**

Information to include when describing an intervention and the location of the information

| **Item number** | **Item** | **Where located **** | |
| --- | --- | --- | --- |
|  |  | Primary paper  (page or appendix  number) | Other ^†^ (details) |
|  | **BRIEF NAME** |  |  |
| **1.** | Provide the name or a phrase that describes the intervention. | Step 4 | Move your way during the W@H day |
|  | **WHY** |  |  |
| **2.** | Describe any rationale, theory, or goal of the elements essential to the intervention. | page 1,2,3; Step 1 results | The trend to work at home has continued since the Covid-19 pandemic. Working at home appears to exacerbate the already elevated levels of unhealthy occupational sedentary behaviour. There is a need to support employees to move more while working at home, taking into account individual, environmental and cultural factors. The aim of this study was to outline the comprehensive development of an intervention to support employees to move more when working at home utilising the novel integration of the Six essential Steps for Quality Intervention Development (6SQuID) and Behaviour Change Wheel (BCW) intervention development frameworks. |
|  | **WHAT** |  |  |
| **3.** | Materials: Describe any physical or informational materials used in the intervention, including those provided to participants or used in intervention delivery or in training of intervention providers. Provide information on where the materials can be accessed (e.g. online appendix, URL). | Step 4 results | The intervention is presented as a toolkit and can be accessed here: <https://actify.org.uk/pahrcw@h>  The toolkit consists of information, strategies, suggestions and resources to help employees reduce sedentary behaviour while working at home. |
| **4.** | Procedures: Describe each of the procedures, activities, and/or processes used in the intervention, including any enabling or support activities. | Step 5 methods  Step 4, 5 results | The toolkit has five modules: 4 targeting employees and 1 targeting managers and leaders. The material presented in the toolkit align with the programme theory. The toolkit can be delivered in a variety of ways depending on the organisation. An example of toolkit delivery is provided in step 5, where 4 modules of the toolkit were tested with Scottish Government employees. To raise awareness of the toolkit and evaluation, an online webinar was hosted using MS Teams, attended by Staff from the Population Health Directorate of The Scottish Government. The toolkit was also promoted through the Saltire blog (an internal blog for The Scottish Government staff). The intervention was delivered through four email newsletters to employees (using DotDigital) which were aligned with the toolkit strategies identified, with each newsletter following a weekly theme. |
|  | **WHO PROVIDED** |  |  |
| **5.** | For each category of intervention provider (e.g. psychologist, nursing assistant), describe their expertise, background and any specific training given. | Step 4, 5 results  Step 5 methods | The intervention is presented as an online toolkit. The delivery of the toolkit depends on the organisation. An example of toolkit delivery is provided in step 5, where 4 modules of the toolkit were tested with Scottish Government employees. In this example, the intervention project team conducted the webinar and developed the blog to promote the toolkit and the project. The team also delivered the intervention through four email newsletters to participants (using DotDigital). |
|  | **HOW** |  |  |
| **6.** | Describe the modes of delivery (e.g. face-to-face or by some other mechanism, such as internet or telephone) of the intervention and whether it was provided individually or in a group. | Step 5 methods  Step 4, 5 results | The intervention is presented as an online toolkit. The toolkit can be accessed by employees individually while working from home. The employees can adopt the strategies and use resources that suit them to help reduce sedentary behaviour while working from home.  The toolkit also has a module for managers and leaders, which can be accessed by line managers and those who have influence over occupational health and wellbeing. |
|  | **WHERE** |  |  |
| **7.** | Describe the type(s) of location(s) where the intervention occurred, including any necessary infrastructure or relevant features. | Step 5 methods  Step 4, 5 results | The toolkit can be accessed online by employees, managers and leaders.  The toolkit was tested with employees from the Scottish Government (Active Scotland Division). The suggestions and strategies in the toolkit can be adopted by employees and managers to suit their environments and infrastructure. |
|  | **WHEN and HOW MUCH** |  |  |
| **8.** | Describe the number of times the intervention was delivered and over what period of time including the number of sessions, their schedule, and their duration, intensity or dose. | Step 5 methods  Step 4, 5 results | The intervention was delivered over a 4-week period, through four email newsletters to employees (using DotDigital) which were aligned with the toolkit strategies identified, with each newsletter following a weekly theme. |
|  | **TAILORING** |  |  |
| **9.** | If the intervention was planned to be personalised, titrated or adapted, then describe what, why, when, and how. | Step 5 methods  Step 4, 5 results | The online toolkit is designed to reflect the theory of change and address factors influencing sedentary behaviour. It comprises strategies, suggestions and resources that employees and managers can adopt depending on their circumstances and contexts. |
|  | **MODIFICATIONS** |  |  |
| **10.^ǂ^** | If the intervention was modified during the course of the study, describe the changes (what, why, when, and how). | Step 5 methods  Step 4, 5 results | Initially, the toolkit consisted of 4 modules designed to support employees. A fifth module was added directed at managers and leaders to create a supportive organisational culture and help them support employees reduce sedentary behaviour. |
|  | **HOW WELL** |  |  |
| **11.** | Planned: If intervention adherence or fidelity was assessed, describe how and by whom, and if any strategies were used to maintain or improve fidelity, describe them. | Step 5 methods and results | Four modules of the toolkit were delivered to employees from the Scottish Government (Active Scotland Division) to test acceptability and feasibility. Data were collected on staff engagement; specifically, attendance at the webinar, views of the Saltire blog, sign-ups to the intervention, and the percentage of recipients who interacted with the newsletter. Additionally, evaluative feedback information was collected at the end of each week, and at the end of the project from all those who signed up to participate. |
| **12.^ǂ^** | Actual: If intervention adherence or fidelity was assessed, describe the extent to which the intervention was delivered as planned. | Step 5 results | The toolkit wad delivered as planned and was feasible to deliver. |

** **Authors** - use N/A if an item is not applicable for the intervention being described. **Reviewers** – use ‘?’ if information about the element is not reported/not sufficiently reported.

† If the information is not provided in the primary paper, give details of where this information is available. This may include locations such as a published protocol or other published papers (provide citation details) or a website (provide the URL).

ǂ If completing the TIDieR checklist for a protocol, these items are not relevant to the protocol and cannot be described until the study is complete.

* We strongly recommend using this checklist in conjunction with the TIDieR guide (see *BMJ* 2014;348:g1687) which contains an explanation and elaboration for each item.

* The focus of TIDieR is on reporting details of the intervention elements (and where relevant, comparison elements) of a study. Other elements and methodological features of studies are covered by other reporting statements and checklists and have not been duplicated as part of the TIDieR checklist. When a **randomised trial** is being reported, the TIDieR checklist should be used in conjunction with the CONSORT statement (see [www.consort-statement.org](http://www.consort-statement.org)) as an extension of **Item 5 of the CONSORT 2010 Statement.** When a **clinical trial** **protocol** is being reported, the TIDieR checklist should be used in conjunction with the SPIRIT statement as an extension of **Item 11 of the SPIRIT 2013 Statement** (see [www.spirit-statement.org](http://www.spirit-statement.org)). For alternate study designs, TIDieR can be used in conjunction with the appropriate checklist for that study design (see [www.equator-network.org](http://www.equator-network.org)).
